# Early-Life Behavioral Time Budgets of a Local Dairy Sheep Breed in Indoor and Pasture Systems

**DOI:** 10.3390/ani16050816

**Published:** 2026-03-05

**Authors:** Silvia Parrini, Valentina Becciolini, Riccardo Bozzi, Francesco Sirtori, Maria Chiara Fabbri, Sebastian Schweizer, Carolina Pugliese

**Affiliations:** Department of Agriculture, Food, Environment and Forestry, University of Florence, 50145 Florence, Italy; silvia.parrini@unifi.it (S.P.); carolina.pugliese@unifi.it (C.P.)

**Keywords:** Massese sheep, grazing, indoor system, age, weaning, lambs, behavior

## Abstract

Given the limited knowledge of early-life behavior in the Massese dairy breed, this study investigated lamb behavior from 5 to 70 days under two rearing conditions to identify age-related behavioral phases potentially relevant to weaning. Twenty-two Massese lambs were reared either in an Indoor housing system during the winter or in an outdoor Pasture system during the spring, in accordance with traditional seasonal management practices. Lambs in both systems remained with their dams with continuous access to milk. Behavioral variables, recorded weekly during daylight and expressed as relative frequencies, were analyzed against age and rearing system, using THI as covariate. Growth influenced most behaviors, with similar age-related trends across rearing systems. Suckling behavior decreased significantly with age, tending to cease at approximately 40 days in Indoor lambs and 50 days in Pasture lambs. Grooming also declined over time, indicating reduced affiliative interactions irrespective of the rearing system. In contrast, solid feed intake and rumination increased progressively, reflecting a transition toward nutritional independence. Pasture lambs engaged in moving and grazing activities while indoor lambs spent most of their time lying. These results suggest preliminary insights into a management context-associated, behavior-based weaning window in Massese lambs.

## Simple Summary

Knowledge about the early-life behavior of Massese sheep, a traditional Italian dairy breed, is still limited. This study aimed to describe how lamb behavior changes during the first two months of life when animals are raised in two common farming systems: Indoor housing and outdoor Pasture. Twenty-two lambs, staying continuously with their mothers and having free access to milk and solid feed, were observed regularly, and behaviors such as suckling, feeding (hay or concentrate, grazing), ruminating, resting, moving, and grooming were recorded over time. Results showed that as lambs grew, suckling and grooming gradually decreased, while eating solid food and ruminating increased, indicating a natural progression toward independence from the mother. The age at which suckling tended to stop differed between farming systems: 40 days in Indoor and 50 days in Pasture lambs. Lambs raised on Pasture spent more time moving and grazing, whereas indoor lambs spent more time resting. These findings represent a first step towards the identification of a behavior-based weaning window in Massese lambs.

## 1. Introduction

In central Italy, the region of Tuscany accounts for approximately 5% of the national sheep population, with over 85% of flocks primarily raised for milk production. Among the traditional dairy breeds reared in this area, the Massese sheep (14,487 heads) represents a key local genetic resource, well-adapted to marginal territories and widespread in mountainous and hilly areas. Primarily classified as dairy ewes, they are recognized for their dual-purpose aptitude both for milk and meat production; however, they are linked to niche products, e.g., traditional cheeses transformed directly at the farm. Massese sheep are characterized by a traditional semi-extensive rearing system following a farming model based on the seasonal availability of natural resources throughout the year. Grazing remains the main source of foraging [1] in the spring and summer periods, while winter rearing is almost entirely carried out indoors. Therefore, the pastoral system is characterized by an inseparable link between season and management.

However, in central Italy, the use of direct grazing Pastures has declined in the last decade, particularly in small-scale sheep farming. This trend is attributed to multiple factors, including increased wolf predation, land abandonment, and socio-economic constraints [2]. Changes in the use of Pasture not only affect nutrient intake but also influence the way animals interact with available feed resources. As a response, animals can adapt and adjust their feeding behavior, based on their experience and environmental context [3,4]. Moreover, García-Favre et al. [5] noted the importance of sheep diet preferences in relation to nutritional needs, diurnal rhythms, and pasture characteristics. Indeed, in pastoral systems, environmental changes play a crucial role in the responses of animals’ behavior. However, considering the typical rearing system of local breeds, information is still scarce. Daily time budgets are essential to evaluate how animal behavioral development adapts to specific seasonal and management contexts, as in the case of the Massese ovine breed.

In addition, in traditional rearing systems, such as those involving dairy Massese sheep, lambs are typically not separated from their mothers after birth until their early slaughter (about 30 days of age) and, when reared as replacement ewes, they usually remain with them from late spring to early autumn within a semi-extensive management system, even if in some areas, partial separation is introduced after 20 days, allowing for daytime separation and nighttime nursing. In contrast, intensive systems (as usual, e.g., for the Lacaune breed) often practice early separation within the first 48 h to optimize milk availability for cheese production. This early separation, as lambs transition from maternal milk to reconstituted milk, negatively affects behavior, the endocrine system, and immune responses [6]. This is also recognized by consumers, whose acceptability of animal products is positively affected when provided information on lambs’ welfare, particularly when reared at pasture with mothers [7]. In this framework, investigating a practical weaning window may represent a key element for farmers’ decisions and for preserving local breeds under system-dependent management. The behavioral implications of rearing practices and natural weaning in local Italian dairy breeds remain poorly understood, and specifically for the Massese breed, there are no studies identifying the critical phase of weaning from a behavioral perspective. At the same time, understanding the behavioral traits during the early life of lambs is crucial for defining management strategies that respect species-specific needs and for identifying a behavior-based weaning window contributing to improving housing and management conditions [6,8]. Indeed, behavioral observation during early life stages can provide practical insights into lambs’ adaptability and resilience [8], potentially providing an ethological characterization that could add value to niche local products of the local Massese breed.

To contribute to this field, the present study describes behavioral patterns of Massese lambs observed in a typical rearing system characterized by both grazing and indoor conditions, each embedded within its respective seasonal and environmental context. Focusing on feeding behavior, social interactions, and environmental adaptation during the first nine weeks of life, the study aims to (a) characterize early-life lamb’s behavioral traits of Massese local breed; (b) quantify time budget and age-related trends under typical seasonal contexts; (c) identify a natural and practical weaning window. These insights can support the refinement of sustainable management practices in traditional dairy sheep farming.

Based on these aims, the study hypothesized that behaviors linked to nutritional development (e.g., suckling, rumination, solid feed intake) would be primarily age-driven, whereas behaviors related to activity and space use (e.g., movement, grazing, lying) would vary mainly according to the management context. We further expected seasonal management to influence daily time budgets, leading to context-specific behavioral adaptations.

## 2. Materials and Methods

### 2.1. Animals and Treatments

The study was carried out in a commercial farm located at 1000 m a.s.l. (116°42′ E, 43°38′ N) in the Pistoia Apennines, Tuscany, central Italy. This territory is mainly characterized by mountains and is considered a marginal area due to its geographical position and historical development. The study was adapted to the traditional, seasonally dependent rearing system of the Massese breed, enrolling all male lambs born in the autumn–winter season (studied indoors) and all male lambs born in the spring season (studied on Pasture). Consequently, lambs were not randomly allocated to rearing conditions, and the rearing system was inherently linked to season. Therefore, the study provides a characterization of two distinct seasonal management contexts.

No invasive treatments or procedures involving the study animals were applied; therefore, in accordance with European Directive 2010/63/EU and the Italian Legislative Decree No. 26 of 4 March 2014, this experiment did not require ethical approval. Purebred Massese male lambs (n = 22) born from multiparous ewes were selected, excluding twins or unhealthy animals, and identified using colored collars within five days of birth. From birth to day 4, lambs and their dams were housed indoors in individual maternity pens in order to facilitate the maternal bond. Lambs remained together with their dams during all life periods, always having the opportunity to suckle milk. No daily separations ever occurred (neither at night nor from a certain age onwards), because the ewes were not milked during the observational period. From the fifth day, pairs of lambs and dams were assigned to either an indoor housing system (hereafter referred to as the Indoor group) or to an outdoor grazing system (Pasture group), according to the traditional rearing practices based on the season of birth. Each lamb was weighed for a minimum of 3 times by the farmer from the beginning to the end of the observational period. The lambs’ age at weightings for the two groups are reported in Table S3. Lambs and dams were maintained in two groups in total, one for each rearing system, in accordance with their social and biological needs and in compliance with animal welfare regulations. For reasons related to the dimension of the flock and to farm management, subdivision into smaller groups to obtain replicates was not feasible, and each rearing system was represented by a group of lambs. As a consequence, system-level effects cannot be fully separated from unmeasured group-specific factors, which may include seasonal and environmental conditions.

The Indoor group (I) included 10 lambs and their dams, reared in stalls and fed by maternal milk, hay, and a pelleted concentrate supplement. The housing system included a barn with an insulated roof and openings at the top, side walls and large doors that remained open during the day. The I group animals were reared exclusively indoors in a collective free-stall pen with a feeding area and a resting area with straw bedding. Free access to water was guaranteed. The daily feeding routine started with a first distribution of concentrate and hay between 07:20 h and 08:20 h every morning. Hay was accessible to ewes, without restrictions for lambs, and was available ad libitum during daytime. The commercial concentrate was distributed in the morning through a creep feeding system, preventing adult animals from accessing the lambs’ supplement. It was provided at a dose of 200 g per capita and remained available afterwards without further additions. The concentrate was composed of barley, maize, soybean meal, wheat bran, field beans, oatmeal, molasses, alfalfa flour, alfalfa pellets, vitamins, and supplements. Hay and concentrate were sampled three times during the trial: at the start, after 30 days, and finally after 60 days.

The Pasture group (P) included 12 lambs and their respective dams, which grazed outdoors during the day and returned indoors for overnight recovery. The time spent in the shelter (night enclosure) varied and did not follow a fixed schedule during the observation period, covering the time from sunset to sunrise. Grazing animals were fed exclusively by the herbage of natural pasture and hay, without any supplementation beyond suckling milk. Shade was provided by the adjacent woodlands and by open-sided shelters. Free access to water was guaranteed, and hay was available ad libitum during the day. The grazing area of the Pasture group changed weekly, so animals rotationally grazed on the plots of land with their respective mothers. Each plot measured between 3 and 4 ha. The enclosures were fenced using materials and structures designed for predator prevention, while the animals were always accompanied by two guard dogs. Representative areas of the pastures were sampled using exclusion cages of 1 m^2^ at all changes in grazing pasture area. The areas of grazing were natural or naturalized pastures where vegetation is dominated by, on average, 60.2% Graminaceae, 25.5% Leguminosae, and 14.3% other families. The yields of herbage mass available were 2500 kg/ha on average. Hay was sampled three times during the trial, as reported for the Indoor group, while pasture herbage was sampled at every rotation.

Chemical composition was determined for all feed resources (hay, concentrate, and herbage): moisture (by drying up to constant weight of the sample), fat (as ether extract), protein and ash contents [9], and fiber components [10]. The results are reported in Table S1 as descriptive statistics (Supplementary Material File S1).

For both the I and P groups, the sample size was determined by the actual flock dimension of the study farm, specifically the number of ewes lambing in the same season. Since the flock size is consistent with the average of the area, it can be considered representative of local extensive farming systems.

### 2.2. Behavioral and Environmental Observations

In this study, the individual lamb was considered the experimental unit. In situ direct behavioral observations were performed every 7 days to cover the lambs’ developmental period from 5 to 72 days of age; consequently, each lamb was observed at 10 time points during the trial, corresponding to slightly different ages across individuals. Observations were conducted by two trained observers who alternated during the observation periods. A preliminary training period was conducted to align the observers’ records, achieving an inter-observer agreement (>90%) before starting the official sessions. A procedure of familiarization was applied with animals before the trial days in order to avoid observers interfering with the spontaneous animals’ activities, particularly those of ewes. Animals were considered to become familiar with observers when they could remain closed for about 5 min without affecting or modifying their activity. Observers were positioned at various fixed vantage points both inside the stall (I) and the Pasture (P) to minimize disturbance. Focal observations were performed by instantaneous scan sampling at 5 min intervals. In both systems, all animals (12 lambs at Pasture and 10 Indoors) were monitored simultaneously at each time point during the observation day. The daily observation time was limited to daylight hours, and thus varied among the different observation days, ranging from 7 to 15 h per day. On average, each observation day included 135 scan time points (675 min) in the indoor system and 143 scan times points (715 min) in the Pasture system, with all lambs recorded simultaneously at each scan.

All other behaviors, including grooming directed toward mothers or peer pairs (i.e., other lambs), as well as any abnormal behavior, drinking and licking, were recorded for each lamb as continuous events (all-occurrence). The lambs’ health status was evaluated daily by recording eventual diseases, medical treatments, lameness, and pathogen infections.

The description of the observed behavioral traits, according to ethograms considered in previous research [11,12,13], is reported in Table 1.

During the observation days, temperature (T; °C) and relative humidity (RH; %) were measured using a portable data logger (Oregon Scientific Inc., Tualatin, OR, USA). The device was positioned in shaded locations at animal height both inside the pen and in the grazing area. Data were recorded instantly every five minutes and coupled with each behavioral observation. At the same time, the observers evaluated and recorded two variables: the presence or absence of wind (dichotomous variable) and the instantaneous weather conditions (sunny, rainy, or cloudy). Using temperature (T) and relative humidity (RH), the Temperature–Humidity Index (THI) was calculated based on Equation (1) [14]:

(1) THI=(1.8·T+32)−(0.55−0.0055·RH)·(1.8·T−26)

The description of meteorological conditions for each rearing system is reported in Table 2.

### 2.3. Data Manipulation and Statistical Analysis

All data manually annotated by the two observers on paper sheets were transcribed and organized into spreadsheets using Microsoft Excel 2016. The data were compiled into a database, where each record included information about the lamb (age and rearing system), as well as the recorded animal behavior and environmental conditions. Data were manipulated and statistically treated using R version 4.4.0 [15]. Under the assumption that each behavior persisted throughout the entire 5 min interval, the time dedicated by individual lambs to each behavior was computed and expressed in total minutes per observation day. Subsequently, individual frequencies of behaviors (BF) were calculated for each observation day, as reported in the following Equation (2), to account for variations in daylight duration in the observation period:

(2) $${BF}_{ijk}=\frac{{BT}_{ijk}}{{DT}_{jk}}$$

where BF_ijk_ is the relative frequency of i-th behavior of the j-th lamb in the k-th observation day, BT_ijk_ is the time (minutes) dedicated to the i-th behavior by the j-th lamb on the k-th observation day, and DT_jk_ is the total observation time (minutes) conducted on the j-th lamb in the k-th observation day.

Among other behaviors, drinking, licking salt and abnormal behaviors were recorded using the all-occurrences sampling method; however, they were not considered in the statistical analysis and were summarized using descriptive statistics. No health events occurred in the lambs during the observational period; consequently, no exclusion of animals or data was required, even for individual daily sessions. Grooming was recorded using all-occurrences sampling as well, and analyzed as the total number of events recorded during the daytime observation period for each lamb.

The normality and homoscedasticity of the residuals were assessed using the Shapiro–Wilk test and visually evaluated, respectively. The daily relative frequency of each behavior was modeled using linear and non-linear mixed-effects regressions. Candidate explanatory variables included lamb age (Age, expressed in days) and THI, both treated as continuous covariates, as well as rearing system (R), treated as a categorical variable with two levels (Indoor and Pasture). Age, THI, and R entered the models as fixed effect terms, while lamb identity (LambID) nested within R was treated as the random effect to account for repeated measurements. Further, the interaction between the R and Age was tested. Model selection was performed using stepwise backward elimination of non-significant terms. The Akaike Information Criterion (AIC) was used as the primary metric for model comparison. When the difference in AIC between two competing models was less than 2, the final model was selected based on the graphical diagnostic of residuals and on the Bayesian Information Criterion (BIC), favoring the most parsimonious model. For each behavioral variable, alternative functions were tested, including linear, quadratic, and higher-order polynomials, as well as non-linear functions as exponential and logarithmic.

In the case of suckling milk, where linear or quadratic models did not completely fit the data, a non-linear mixed-effects model was applied (Equation (3)) using the nlme function of the nlme package in the software R (version 4.4.0). Specifically, this behavior pattern was better described by a negative exponential function:

(3) $$Y_{ijk}=[(a_{k}+u_{ai})+\alpha_{k}\cdot {THImed}_{k}]\cdot {exp}^{-[\left(b_{k}+u_{bi}\right)+\beta_{k}\cdot {THImed}_{k}]\cdot {Age}_{ij}}+\varepsilon_{ijk}$$

where

Y_ijk_ = relative frequency of behavior for lamb _i_, in rearing system _k_, at observation _j_;

a_k_, b_k_ = intercept and decay rate parameters within rearing system _k_;

α_k_, β_k_ = coefficients capturing the effect of THI on intercept and decay rate within rearing system _k_;

u_ai_, u_bi_ = random effect on a_k_ and b_k_ of lamb _i_;

THImed_k_ = medium THI for all lambs and all observations, within rearing system _k_;

Age_ij_ = age (days) of lamb _i_, at observation _j_, included in the model as a covariate;

ε_ijk_ = residual error.

For normally distributed behavioral data, specifically solid feeding and lying, records were analyzed using the following mixed models fitted using lmer function of the lme4 package in the software R (version 4.4.0). The model for solid feeding included log-transformed Age as an explanatory variable (Equation (4)):

(4) $$Y_{ijk}=\beta_{0}+{\beta_{1}\cdot R}_{k}+\beta_{2}\cdot log(Age_{ij}+1)+{\beta_{3}\cdot (R_{k}*log(Age}_{ij}+1))+u_{k(i)}+\varepsilon_{ijk}$$

where

Y_ijk_ = relative frequency of behavior for lamb _i_, observation _j_, and rearing system _k_;

β_0_ = overall intercept;

β_1_ = regression coefficient for the rearing system;

β_2_ = regression coefficient for the logarithmic effect of age;

β_3_ = regression coefficient for the interaction between the rearing system and the logarithmic effect of age;

R_k_ = fixed effect of the rearing system (two levels: Indoor and Pasture);

Age_ij_ = age (days) of lamb _i_ at observation _j_, included in the model as a covariate;

Age_ij_ + 1 = age of lamb i at observation j, adjusted by adding 1 to allow logarithmic transformation of zero values.

u_k(i)_ = random effect of lamb _i_ nested within rearing system _k_;

ε_ijk_ = residual error term.

The model for lying behavior was fitted according to Equation (5):

(5) $$Y_{ijk}=\beta_{0}+{\beta_{1}\cdot R}_{k}+{\beta_{2}\cdot Age}_{ij}+\beta_{4}\cdot {{Age}^{2}}_{ij}+\beta_{5}\cdot {THI}_{ijk}+u_{k(i)}+\varepsilon_{ijk}$$

where:

Y_ijk_ = relative frequency of behavior for lamb _i_, observation _j_, and rearing system _k_;

β_0_ = overall intercept;

β_1_ = regression coefficient for the rearing system;

β_2_ = regression coefficient for the effect of age;

β_4_ = regression coefficient for the quadratic effect of age;

β_5_ = regression coefficient for the THI effect;

R_k_ = fixed effect of the rearing system (two levels: Indoor and Pasture);

Age_ij_ = age (days) of lamb _i_ at observation _j_, included in the model as a covariate;

THI_ijk_ = Temperature–Humidity Index, included in the model as a covariate;

u_k(i)_ = random effect of lamb _i_ nested within rearing system _k_;

ε_ijk_ = residual error term.

For rumination, moving, and standing (normally distributed), a linear mixed model was applied (Equation (6)), using lmer function of the lme4 package in the software R (version 4.4.0):

(6) $$Y_{ijk}=\beta_{0}+{\beta_{1}\cdot R}_{k}+{\beta_{2}\cdot Age}_{ij}+\beta_{5}\cdot {THI}_{ijk}+u_{k(i)}+\varepsilon_{ijk}$$

where

Y_ijk_ = relative frequency of behaviors for lamb _i_, observation _j_, and rearing system _k_;

β_0_ = overall intercept;

β_1_ = regression coefficient for the rearing system;

β_2_ = regression coefficient for the effect of age;

β_5_ = regression coefficient for the THI effect;

R_k_ = fixed effect of the rearing system (two levels: Indoor and Pasture);

Age_ij_ = age (days) of lamb _i_ at observation _j_, included in the model as a covariate;

THI_ijk_ = Temperature–Humidity Index, included in the model as a covariate;

u_k(i)_ = random effect of lamb _i_ nested within rearing system _k_;

ε_ijk_ = residual error term.

The count of grooming behavior towards other lambs and dams was analyzed with a Zero-Inflated Poisson model, implemented via the glmmTMB package in the software R (version 4.4.0). The model defines the count Y_ijk_ as following a distribution with two components: a Bernoulli distribution modeling the probability π that Y_ijk_ is equal to zero and a Poisson (λ_ijk_) distribution with probability (1 − *π*) for non-zero counts. The probability π of observing a zero count was modeled using a logit link function on a constant intercept (α_0_), as in Equation (7):

(7) $$logit\left(\pi \right)=\alpha_{0}$$

The expected mean rate λ_ijk_ for the non-zero counts was modeled using a log link function including both fixed and random effects (Equation (8)):

(8) $$\log\left(\lambda_{ijk}\right)\;=\beta_{0}+\beta_{1}\cdot R_{k}+\beta_{2}\cdot Age_{ij}+\beta_{5}\cdot THI_{ijk}+u_{ki}$$

where

λ_ijk_ = is the expected count for observation _i_, rearing system _j_, and lamb _k_;

β_0_ = overall mean of the observation (intercept);

β_1_ = regression coefficient for the rearing system;

β_2_ = regression coefficient for the effect of age;

β_5_ = regression coefficient for the THI effect;

R_k_ = fixed effect of the rearing system (two levels: Indoor and Pasture);

Age_jk_ = age (days) of lamb _k_ at observation _j_, included as a continuous covariate;

u_k(i)_ = random effect of lamb _i_ nested within rearing system _k_;

THI_ijk_ = fixed effect of the Temperature–Humidity Index.

The fitted model included the variable THI only for grooming towards others, while for grooming towards the dam the only retained variables were R, Age and LambID. Finally, to model lamb growth, the following linear mixed model was fitted (Equation (9)) using lmer function of the lme4 package in the software R (version 4.4.0):

(9) $$Y_{ijk}=\beta_{0}+\beta_{1}\cdot R_{k}+\beta_{2}\cdot {Age}_{ij}+u_{i\left(k\right)}+\varepsilon_{ijk}$$

where

Y_ijk_ = weight of lamb _i_;

β_0_ = overall intercept;

β_1_ = regression coefficient for the rearing system;

β_2_ = regression coefficient for the effect of age;

R_k_ = fixed effect of the rearing system (two levels: Indoor and Pasture);

Age_ij_ = age (days) of lamb _i_ at observation _j_, included in the model as a covariate;

u_j(k)_ = random effect of lamb _i_ nested within rearing system _k_;

ε_ijk_ = residual error term.

## 3. Results

Behavioral patterns observed in the two rearing contexts are presented below as trends developing across the first two months of life. These patterns reflect the typical seasonal and management conditions of the local breed under which each system was observed. Before detailing behavioral trends, it is important to note that the growth patterns of lambs showed a continuous increase in average body weight (kg) over the experimental period (Supplementary Table S2 and Figure S1). The linear model indicated a significant effect of age, with an estimated average daily gain for lambs amounting to 182 ± 10 g. In the first week postpartum, lambs in group I weighed an average of 7.13 ± 0.83 kg (at 6.3 days of age), while group P lambs averaged 5.10 ± 0.42 kg (at 7.5 days of age). However, lambs showed similar growth patterns whether they were reared indoors or on pasture, and no significant effect of the rearing system was detected, as reported in Supplementary Material File S2.

The behavioral trends were consistent with the age progression, with no significant interactions between the rearing system and lamb age. As shown in Table 3 for suckling milk and Table 4 for the other behaviors, lambs spent different time on each activity mainly depending on age. Trends for all the investigated behaviors are presented by the estimated equations modeling the relative frequency of each behavior as a function of age (days). Separate equations were provided when statistically significant system-specific patterns emerged.

### 3.1. Feeding Behavior

**Figure 1:**
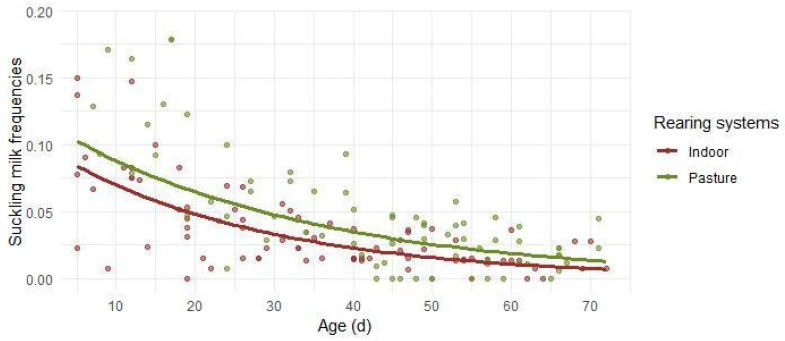
Predicted relative frequencies of suckling milk in relation to lambs’ age in days. Separate lines are reported for the Indoor and the Pasture groups, due to statistical significance. Dots depict the observed frequencies.

The suckling time budget (Figure 1) showed a clear age-related decline, consistent with the exponential decay structure of the model (R^2^_C_ = 0.747). Lambs reported the highest levels of suckling during the earliest days of life, followed by a progressive reduction over time until reaching a minimal plateau. In the Indoor context system, the lambs’ suckling frequency decreased to about 5% of daytime activity by day 20, reaching approximately 2.5% by day 40, after which it tended to stabilize at minimal levels for the remainder of the observation period. Pasture lambs also exhibited a progressive reduction in suckling over time. Suckling frequency declined to around 5% of daytime activity by day 30, and to approximately 2.5% by day 50, followed by stabilization at minimal values thereafter.

**Figure 2:**
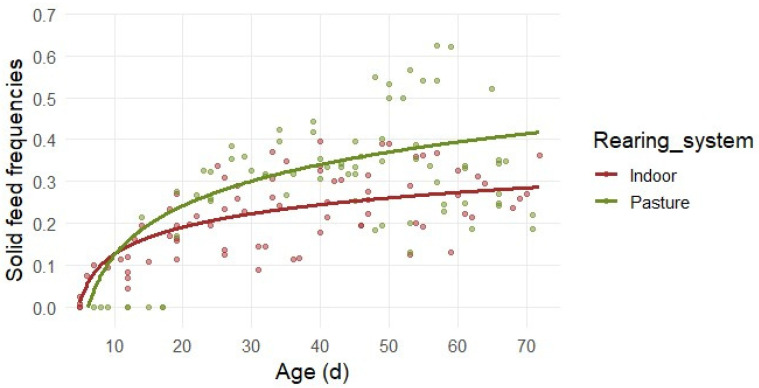
Predicted relative frequencies of solid feeding in relation to lambs’ age in days. Separate lines are reported for the Indoor and the Pasture groups, due to statistical significance. Dots depict the observed frequencies.

Solid feeding showed a non-linear, age-related pattern in both systems (Figure 2) effectively modeled by a logarithmic trend (R^2^_C_ = 0.625). Lambs began to engage with solid feed in early life, starting from zero as expected. The significant interaction between age and the rearing system (β_3_ = 0.055, *p* = 0.0029) indicated that the interest for solid feed differed between the two groups. Indoor lambs initiate solid feed interest at approximately 5 days of age with frequencies near zero, followed by a sharp initial increase in interest during the first 20 days and a more gradual progression that tends to stabilize at 26–29% of total time achieved among 40 to 70 days. Pasture lambs began to engage with solid feed after 10 days of age; however, this group showed a consistent and marked increase in interest throughout the observation period. By 50–60 days of age, feeding frequencies in the Pasture group reached values of 38–40%, and increased to 42% of engaged time at 70 days. This divergence, supported by the significant interaction between age and rearing system (*p* = 0.002), suggests that the pasture environment promotes a rapid and intensive behavioral transition toward forage consumption.

**Figure 3:**
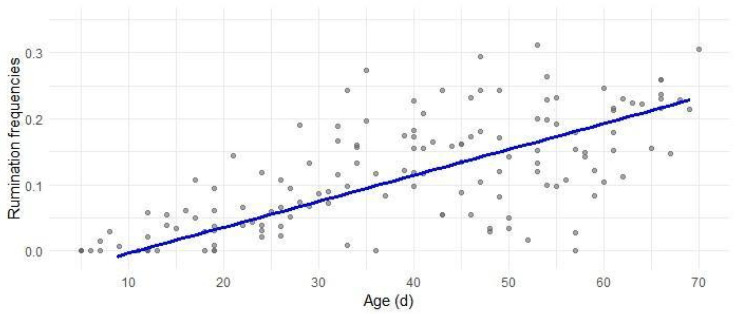
Predicted relative frequencies of rumination in relation to lambs’ age in days. Gray dots depict the observed frequencies; the blue line is the regression line estimated by the model in Equation (6).

The model for rumination behavior (Figure 3) described the time spent by lambs chewing, re-mastication, regurgitation, and re-deglutition of feed across the first months after birth (R^2^_C_ = 0.674). The absence of difference between systems indicate that rumination followed the same pattern in both rearing systems, with a consistent chronological trend regardless of the specific environmental context. The results showed a clear, significant and linear increase in rumination time with age (β_2_ Age = 0.0039, *p* < 0.001), indicating a progressive development of this activity throughout the growth period. Lambs started to ruminate (>0.1) around 9 days, reaching approximately 25% of total daytime rumination by 70 days of age.

### 3.2. Dynamic and Static Behavior

**Figure 4:**
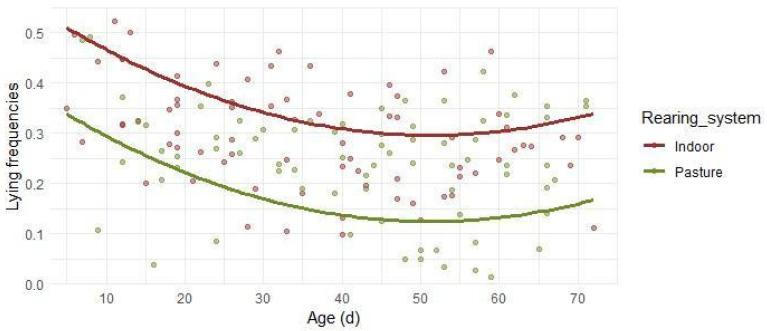
Predicted relative frequencies of lying in relation to lambs’ age in days. Separate lines are reported for the Indoor and the Pasture groups, due to statistical significance. Dots depict the observed frequencies.

Lying behavior (Figure 4) was characterized by a high initial level, linked to the first days of observations within 10 days of age. Over time, lying showed a non-linear age-related pattern (R^2^_C_ = 0.629), with a decrease during the first part of the growth period (with the lowest percentages of time between 45 and 55 days of age), as indicated by the negative linear effect (β_3_ Age = −0.0102, *p* < 0.001) and the positive quadratic effect of age (β_4_ Age^2^ = 0.0001, *p* < 0.001). In the Indoor system, lambs devoted about 50% of the total time to lying within the first 10 days of age, with the lowest percentages of time corresponding to about 30% between 45 and 55 days of age, followed by a slight increase, remaining within approximately 35% of the time budget. In Pasture lambs, lying accounted for about 30–35% of the total time in the first 10 days of age, while over time, lying reached the lowest percentages of 12%, with a slight increase to around 15% at the end of the observation period.

**Figure 5:**
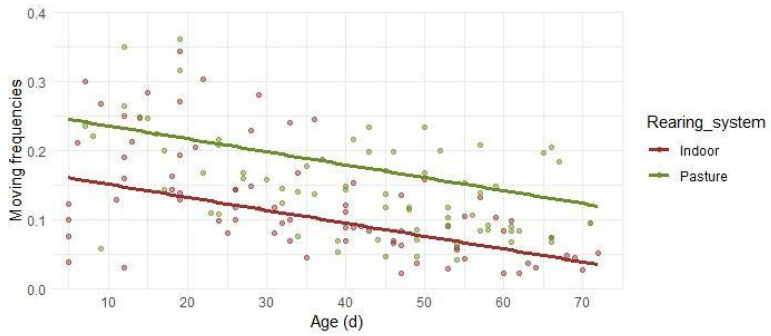
Predicted relative frequencies of moving in relation to lambs’ age in days. Separate lines are reported for the Indoor and the Pasture groups, due to statistical significance. Dots depict the observed frequencies.

Moving behavior (Figure 5) showed an age-related decline in both systems, characterized by a gradual reduction as lambs aged (R^2^_C_ = 0.487). The model parameters indicated a significant linear effect of age (β_2_ Age = −0.0019, *p* < 0.001), describing a progressive decrease with the lambs’ growth and reflecting a shift from early exploratory activity towards more stable behavioral routines. Indoor lambs, in the first days of life and up to 10 days of age, moved for about 15% of the total time budget. Movement then declined gradually, reaching around 10% at 40 days, and dropping below 5% from 65 days of age to the end of the observation period. In Pasture lambs, movement started at approximately 25% in the earliest observations, reaching about 20% at 30 days, and falling below 15% from 55 days onward.

Standing time, defined as periods in which lambs were not in motion (standing or inactive), represents a consistent, baseline component of the lambs’ daily routine. According to the model (R^2^_C_ = 0.281), this behavior was not influenced by the rearing system or age progression (*p* > 0.05), with an overall estimated mean of 13.1 ± 0.8% of the daytime budget (95% CI: 11.4–14.8%).

### 3.3. Other Behaviors

No abnormal behaviors, such as stereotypies or aggressive patterns, were observed among the study lambs and were therefore not reported in the results.

Among other behaviors, drinking, playing, and salt licking were reported as event frequencies (bouts per day) relative to the all-occurrence recording method. These behaviors were standardized to a fixed 8 h daily observation window to ensure consistency and were summarized using descriptive statistics only, as they were not included in the main inferential models.

Drinking water occurred at an average frequency of 1.6 ± 1.2 bouts/day (observed in 18% of lambs) while salt licking occurred at an average of 1.0 ± 0.2 bouts/day (observed in 32% of lambs). All lambs (100%) displayed playing behaviors, with the dam and other lambs, as well as solitary play with an average event of 5.5 ± 5.4 bouts/day.

**Figure 6:**
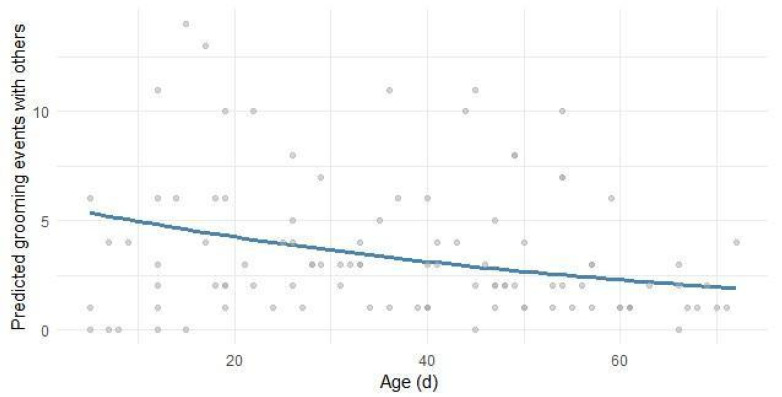
Predicted count (nr. of events per day) of lambs’ grooming towards other co-specifics in relation to their age in days. Gray dots depict the observed events; the blue line is the regression line estimated by the model in Equation (8).

Grooming towards other lambs or other ewes of the flock referred to social interactions in which a lamb actively licked or nibbled another individual (Figure 6). A significant decrease in the events of grooming with age was found (β_2_ Age = −0.0105, *p* < 0.001), while no statistical differences emerged between rearing systems (R^2^_C_ = 0.356). These results highlighted an age-related decline in social interactions, independent of rearing systems.

**Figure 7:**
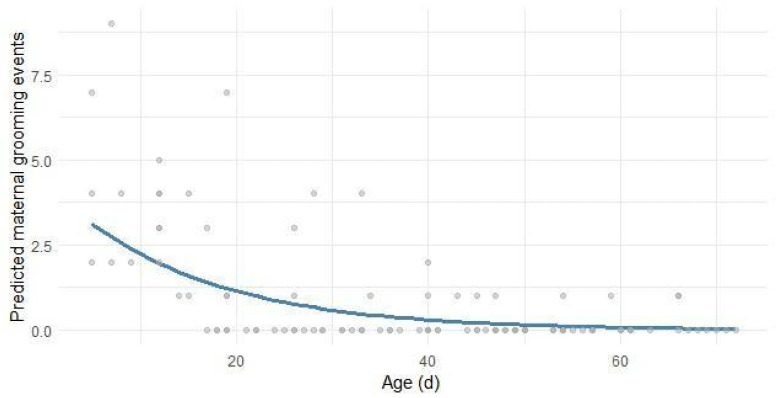
Predicted count (nr. of events per day) of lambs’ grooming towards their dams in relation to their age in days. Gray dots depict the observed events; the blue line is the regression line estimated by the model in Equation (8).

Grooming towards dams, as affiliative interactions initiated by lambs such as licking or gentle nuzzling, is reported in Figure 7. The results revealed a clear and significant decline in the occurrence of these events over time (β_3_ Age = −0.0713, *p* < 0.001), indicating that maternal-directed grooming was predominantly expressed during the earliest stages of life and gradually diminished as the lambs aged. No other variables, including rearing system or THI, were included in the model, suggesting that age was the primary driver of this behavioral shift (R^2^_C_ = 0.801).

### 3.4. Effects of Environmental Conditions

Finally, the Temperature–Humidity Index (THI) was included in all behavioral models as a proxy related to the seasonal context rather than as an indicator of heat stress. THI values averaged 56 in the Indoor system and 70 in the Pasture system, both within the thermal comfort zone for lambs, even if higher values were reached in the grazing system. However, higher THI values were associated with small adjustments in behavior. For example, suckling (β_I_ = −0.0048, β_P_ = 0.0057), rumination (β_5_ = −0.0023), and movement (β_5_ = −0.0031) showed minor variations with THI, while lying increased slightly at higher THI levels. Solid feeding and grooming were not affected. To conclude, observed THI differences likely reflect the seasonal and environmental context inherent to each system.

## 4. Discussion

In the context of the Massese breed and, more broadly, of semi-extensive dairy sheep farming in central Italy, this study contributed to the knowledge of natural behavior patterns of lambs during early growth, with particular focus on the transition from milk suckling to solid feed interest, under two different livestock contexts which include seasonal and environmental conditions, both commonly adopted in marginal and mountainous areas of Italy. While acknowledging the limited number of experimental units, constrained by the operational limits of a representative local farm, the findings should be considered a baseline for the specific conditions studied, while further research on larger scales could help confirm the broader applicability of these trends across different Mediterranean farming environments.

The growth of the lambs was found to be the same for both rearing systems and was primarily affected by age. No detrimental effects were detected in the behavioral parameters: stereotypic and aggressive behaviors were not recorded, although their absence cannot be interpreted as definitive evidence that they did not occur, as rapid and instantaneous events require evaluation using dedicated protocols.

The suckling time-budget and interest in milk progressively declined over time in both rearing systems, highlighting that the weaning process in sheep progressed naturally; however, milk consumption still persisted at extremely low levels, close to zero, beyond two months of age. In Indoor-reared lambs, the decline in suckling behavior, reaching the 5% threshold by day 20 and the 2.5% threshold by day 40, may reflect an earlier transition to solid feed use. Freitas-de-Melo [16] in Santa Ines × Dorper crossbred lambs suggested that free access to creep feeding progressively increased nutritional independence from their mothers through reduced suckling time and increased concentrate intake. Contrary to this, a study suggested that lambs reared with their dams maintained suckling up to 100–130 days of age [17]. Previous literature [18] indicated that the reduction in suckling is not driven exclusively by the lamb’s behavior but is often initiated by the mother ewe. Fonsêca et al. [18] observed that the ewe refusal behavior primarily limited suckling attempts in the first 20 days post-lambing, followed by a decline in the lamb’s initiative at 40 days. Similarly, Dwyer and Lawrence [19] demonstrated that ewes tend to progressively restrict access to the udder during natural weaning. Previous research on the Massese breed [20] suggested that the maternal investment was evident above all in the early stage of lactation. Indeed, through prolonged nursing and maternal instinct at pasture, ewes could deliberately keep their lambs close to protect them from natural hazards. These behaviors could be further investigated to better understand the ewes’ perspective. In Pasture-raised lambs, a sustained dependence on milk was maintained at all ages, reflecting both a natural mother–offspring dynamic and a slower transition to solid feed. The decline in suckling activity reached a first threshold (5%) at 30 days and a second (2.5%) at 50 days, in line with Fonsêca et al. [21] who reported a similar trend in Santa Ines and Dorper lambs (0 to 63 days) with access to pasture. Thorhallsdottir et al. [22] observed that lambs developed a preference for grazing over milk between 28 and 56 days of age, learning from their mothers, which aligns with early weaning. The information emerging from the study of suckling behavior may be useful as a preliminary behavioral-based indicator of the natural weaning window in the Massese breed, which appears to be 40 and 50 days for Indoor- and Pasture-reared lambs, respectively. Furthermore, results suggested that the environmental context (ranging from external stimuli to different feed resources) plays a key role in modulating not only the initial intensity of suckling but also its temporal dynamics. This implication is consistent with the idea that the rearing environment can stimulate or limit specific behaviors. Finally, although the Massese breed typically reports a peak in milk production during the early lactation stage, it is possible to hypothesize that with a delayed weaning, the additional milk from the ewe could still be used for human consumption with reduced ethical concerns. In livestock terms, recent studies have indicated that during the late lactation period, specifically the last 30 days, a ewe can produce approximately 25% to 30% of its total yield [23]. Further research could aim to assess the productive and economic feasibility of delayed weaning in local dairy sheep breeds.

Solid feeding behavior started from total absence at birth, progressively increased with the lamb’s age, reflecting a growing interest in solid feed; from a physiological perspective, this increase in time spent feeding aligns with the growth of lambs and their rising nutritional requirements. Notably, while the suckling curve follows an exponential decay, solid feeding increases according to a logarithmic trend, suggesting a compensatory mechanism. In Indoor lambs, the interest in solid feed began very early, likely driven by exploratory behavior and curiosity, considering the low time investment initially observed, but achieved an interest plateau at about 29% of their time at 70 days. This underlines the importance of providing solid feed from the very beginning of the rearing period to support this natural exploration phase. Regarding the Pasture context, interest in solid feed began later than in Indoor lambs; however, the slope of the curve indicates a steeper subsequent increase in interest (up to 42% at 70 days). Alvarez-Rodriguez et al. [24] reported that Churra lambs reared on pasture with their own dams began grazing later than observed in our study, specifically at three weeks of age, although they confirmed that time spent grazing increased regularly. The presence of dams probably affected the interest in solid feed, as cognitive processes are involved in how ruminants learn to graze, as reported by Garrett et al. [25] and Faisca et al. [26]. Youssef et al. [27] found that pre-weaning grazing experience had a greater effect on weaned lamb grazing behavior than social facilitation (i.e., the presence of experienced adult animals). This trend was also observed by Fonseca et al. [18], even though in semi-extensive systems with Morada Nova lambs, they observed higher frequencies at all ages (54% at 20 days, 58% at 30 days, and 55% at 40 days) compared to those recorded in our study. Also, Alvarez-Rodriguez et al.’s [24] research on lambs reared with their dams suggested a higher grazing time (55.2% as average) of daylight hours during the recording period compared to our P lambs at all ages. Indeed, other studies even reported higher grazing times for adult sheep, exceeding 65%, as observed by Bojkovski et al. [28] in grassy paddocks, indicating that grazing activity continues to increase in adulthood. In this context, it has been reported in the literature that sheep in temperate regions conduct 65% to 100% of their grazing activity during daylight hours, also suggesting a tendency to avoid grazing in the dark to reduce the risk of predation and conserve energy [29]. Finally, the significant interaction between the age and rearing system suggests that the pasture context acts as a behavioral stimulus, which can be interpreted as a proactive dietary adaptation strategy. The availability and type of feed are likely key factors influencing feeding behavior. In pasture environments, continuous access to fresh herbage and natural feeding resources may enhance feeding activity. Garrett et al. [25] reported that offering diets with flavor diversity compared with a repetitive diet resulted in higher dry matter intake, specifically for fresh forages. Freitas-de-Melo et al. [16] also reported that the greater time spent in a feeding area could be affected by stronger gregariousness patterns among lambs that were more exposed to external environmental stimuli.

Rumination behavior showed a clear linear increase with age, consistent with the physiological development of the ruminal system and with other feeding behavior. This progressive rise reflects the natural maturation of the digestive function and the increasing efficiency in processing solid feed. Notably, the single curve describes rumination across both rearing systems, and the absence of differences suggested that rumination is a fundamental and universal behavior, firstly driven by internal developmental processes. It should be considered that both groups have mothers’ milk availability and forage resources; therefore, nutritional maturation followed the same pattern. Khan et al. [30] highlighted that, both in natural and pastoral systems, the combination of milk and forage resources, especially pasture, provides the necessary stimuli for the proper development and growth of young ruminants, including the ruminal apparatus. Even though it is known that rumination behavior and its duration are influenced by the availability and particle size of solid feed, similarly to our study, Freitas-de-Melo et al. [16] reported that the rumination time did not differ between the two considered feeding systems, reinforcing the idea that at this phase, rumination is closely linked to age-related physiological changes. The lambs’ rumination achieved about 22% of the diurnal time at 72 d, which is anyway lower than rumination time in adult animals. For sheep, the time spent ruminating is estimated to be about one-third of the day, ranging from 4 to 9 h, divided into 15 to 20 short periods throughout the day [31].

Regarding the movement behavior, the decline with the growth of animals reflected the natural behavioral shift from exploration and play to more feeding and digestion-related activities during growth. The inverse relationship between movement and growth may be partly explained by the behavioral dynamics of early life, where lambs tend to follow their dams in search of maternal protection [32]. The ewe–lamb bond involves not only suckling but also maintaining close physical and visual contact. In this context, previous studies have reported that during early lactation, grazing time in lactating ewes is 7–12% higher compared to non-pregnant sheep [33]. These ewes tended to move more while searching for feed, which may explain the higher levels of movement observed in young lambs, who followed their dams to maintain proximity. This following of dams’ behavior may therefore have contributed to the elevated movement time recorded during the initial life stages in our study, before gradually declining as the lambs became more independent. Nevertheless, Fonsêca et al. [18] did not highlight a change in time spent moving when the lambs aged, even if their observations were exclusively referred to playing. For the Indoor lambs, the rapid reduction in movement (from 15% to 5% after 65 days) reflected the confined system, suggesting that digestive maturity combined with environmental saturation could affect movement interest due to localized, predictable resources. In Pasture lambs, the prolonged movement levels (remaining above 15% even in the later stage) were likely sustained by the necessity for autonomous foraging and the continuous interaction with environmental stimuli [34], such as the weekly change in paddocks [35].

Lying behavior followed a non-linear trend, with an initial decline during the early stages of life, followed by a tendency to stabilize in the range between 45 and 55 days of age. This pattern suggests that rest requirements progressively decrease as lambs grow, until reaching a more stable resting routine. According to our study, the greater amount of time that animals spend in solid feed inadvertently coincides with less time inactive, as also reported for different grazing animal species [12,36]. The lying time observed in Indoor lambs—which initially accounted for 50% and later stabilized around 35% of the time budget (with a minimum of 30% between 45 and 55 days)—is a key component of energy management during early growth. However, these values are notably lower than the 62% reported for adult, housed sheep by Lauber [37]. This difference might also be affected by a greater exploration drive of growing lambs compared to adult ewes. Research [33,38] suggests that the housing conditions affected the locomotor activity, suggesting that animals living in an Indoor confined environment have limited freedom of movement linked to the reduction in ability to perform the behavioral patterns linked to social and feeding behaviors. Pasture lambs exhibited lying times, ranging from 30 to 35% initially down to a stabilization around 15% (with a minimum of 10–15% in the central phase); probably affected by the behavioral priorities of grazing and exploration in an outdoor environment. A free-ranging sheep study [12] reported a high resting budget of 24%, confirming however that this behavior is affected by the age of animals.

Standing behavior remained relatively constant throughout the observation period, with no significant variation detected over time (age) or between the rearing systems included in the study. This lack of significant divergence suggests that standing may represent a baseline postural state in young lambs, which remains relatively shielded from environmental or dietary influences during early development. On the contrary, Pullin et al. [39] reported that the time spent resting followed a trend similar to lying behavior, indicating a possible connection between the two postural states in terms of inactivity.

Grooming behavior showed a significant decline with age, both when directed towards conspecifics and towards the dam. This pattern reflected the nature of grooming as early social and affiliative behavior, which tended to decrease as lambs matured and became more independent. Grooming directed at conspecifics or other ewes in the flock suggests affiliative social interactions, while maternal grooming likely reflects the strength of the ewe–lamb bond during the early postnatal period. A previous study showed that during the first phase of nursing, the lamb–dam relationship is characterized by strong physical attachment, which gradually evolves throughout lactation [17]. Although grooming and maternal behaviors have been widely described in lamb ethograms, comparisons between studies are often limited due to differences in breed, litter size, and reproductive management. For instance, a study [13] reported higher rates of grooming between lambs and their dams (ranging from 5.7% to 6.8% of total time) from birth to weaning (35 days) than those observed in our study. Our results confirmed that this bond began to loosen after the first few weeks of life: grooming directed toward the mother was concluded after approximately 40 days of age in both rearing systems, indicating a natural reduction in mother–offspring bonding, while grooming towards others tended to stabilize at a low number of events at the same age. This decline, occurring after 40 days, marks a critical behavioral turning point: it coincides with the minimal plateau in suckling and the sharp increase in solid feed intake. This threshold suggests a shift in the lamb’s priorities, where energy and time are reallocated from social-affiliative dependence toward the nutritional and metabolic requirements of a developing ruminant.

## 5. Conclusions

In conclusion, the study reflects the typical environmental and production context for local breeds such as the Massese under the two rearing systems as they typically occur in practice, providing insights to support improvements in management strategies.

- Massese lambs’ behavior patterns were primarily influenced by growth. The transition from milk to solid feeding occurs naturally, as evidenced by the progressive decline in suckling behavior and grooming interactions. However, the rearing environment significantly modulated how lambs approached solid feed. While indoor lambs showed an earlier interest, likely driven by exploratory behavior, those on pasture exhibited a sharper subsequent increase in feeding activity despite a slight delay in the initial onset. This suggests that the pasture context acts as a strong behavioral stimulus, probably encouraging a proactive dietary adaptation strategy.
- Daily time budgets followed similar age-related trends across both systems, yet showed distinct intercepts for all behavior (excluded solid feeding). This confirms that while the developmental trend is consistent, the rearing context had an effect on how lambs allocate their time and interact with their environment, reflecting different behavioral priorities.
- The rearing context seems to influence the timing of behavioral transitions. Specifically, the reduction in lambs’ interest in milk observed after 40 days (Indoor) and 50 days (Pasture), may represent adaptive candidate windows for weaning to be tested in future experimental studies. Considering the traditional rearing system of the Massese breed (indoors during winter and outdoors during spring–summer) different potential windows of weaning could be proposed, with reduced implications consistent with the idea that the rearing environment can stimulate or limit specific behaviors.

Future research should explore the ewe’s role in modulating suckling and weaning behaviors in greater depth, as well as the long-term effects of early-life environmental conditions on adult sheep productivity and welfare. On the other side, the detailed insights of this study since such systems are subject to annual variations in climatic conditions, forage availability, and management practices could serve as a foundational basis for future research. To fully validate these natural weaning strategies, further studies should replicate groups across different seasons and farms, integrating measurements of ewe behavior, individual solid intake, and milk yield ideally supported by precision livestock farming. Finally, the valorization of those strategies can help to promote sustainable and ethical production systems, attributes that are increasingly valued by consumers of animal products.

## Figures and Tables

**Table 1:** Description of the behavioral traits observed.

Behavior	Description
Instantaneous Scan Sampling	
Suckling milk	Rhythmic milk intake from the dam’s udder; the lamb is positioned at the udder with a teat in its mouth, showing swallowing and sucking movements.
Solid feeding	Intake of non-liquid feed, further classified by substrate:
	- hay or concentrate feeding: act of picking, masticating, and swallowing hay or pelleted concentrate.
	- grazing: intake of pasture herbage, including the time spent searching for food with the muzzle in close proximity to the sward, whether stationary or during slow locomotion.
Ruminating	Regurgitation, re-insalivation, re-mastication, and re-deglutition while standing, lying, or moving.
Moving	Active locomotion (walking, trotting, running) across the pen or pasture. This category is not associated with foraging, and excludes slow movement where the muzzle remains in contact with the sward.
Standing	Static posture on all four legs with the head held above the shoulder line; the animal is not engaged in any other specific activity.
Lying	Static posture in which the animal is resting on the ground in a ventral or lateral position; limbs are not supporting the body.
All-Occurrences Sampling	
Drinking	The animal is stationary with its muzzle in the water trough, exhibiting active suction and swallowing of liquid.
Licking salt	The animal is stationary, licking or nibbling the mineral salt block.
Grooming	The animal exhibits licking or nibbling movements directed towards other lambs or its dam.
Abnormal behavior	Includes stereotypies (repetitive non-functional movements), aggression (active physical conflict between individuals), and signs of illness (lethargy, coughing, or abnormal posture due to physical distress).

**Table 2:** Descriptive statistics of meteorological conditions registered during behavioral observations in the two rearing systems.

Rearing System		Indoor	Pasture
THI	mean	56.7	70.4
	SD	4.9	7.9
	range	49–69	51–82
Weather conditions (% of observations)	sunny	57.8	62.1
	cloudy	29.1	32.1
	rainy	13.1	5.8
Wind (% of observations)	absence	99	44.7
	presence	1	55.3

**Table 3:** Coefficient estimates with standard errors (S.E.) and *p*-values for the non-linear mixed model fitted to suckling milk frequencies.

Coefficient	Coefficient Estimate	S.E.	*p*-Value
a—Indoor	0.7292	0.1540	<0.0001
a—Pasture	0.4556	0.1893	0.0176
b—Indoor	0.3042	0.0827	0.0003
b—Pasture	−0.3430	0.0951	0.0005
α—Indoor	0.0112	0.0027	0.0001
α—Pasture	0.0090	0.0032	0.0057
β—Indoor	−0.0048	0.0015	0.0015
β—Pasture	0.0057	0.0016	0.0005

**Table 4:** Coefficient estimates with standard errors (S.E.) and *p*-values for behavior models according to the best-fit model (linear, quadratic, or logarithmic).

	S FEED	RUM	MOV	STA	LYI	GR O	GR M
Fixed effects							
**Intercept**							
	Indoor	Overall	Indoor	Overall	Indoor	Indoor	Overall
Estimate	0.0075	0.1015	0.3679	0.3071	0.1465	1.6166	1.5628
S.E.	0.0367	0.0521	0.0661	0.0523	0.0895	0.1466	0.1337
*p*-value	0.8383	0.057	<0.001	<0.001	0.1040	<0.001	<0.001
	Pasture		Pasture		Pasture	Pasture	
Estimate	−0.0947	-	0.4525	-	−0.0252	1.2508	-
S.E.	0.0685	-	0.0232	-	0.0401	0.2219	-
*p*-value	0.1379	-	<0.001	-	<0.001	0.099	-
**Age (β_2_)**							
Estimate	0.0661	0.0039	−0.0019	0.0006	−0.0102	−0.0137	−0.0105
S.E.	0.0097	0.0003	0.0004	0.0003	0.0018	0.0044	0.0030
*p*-value	<0.001	<0.001	<0.001	0.0930	<0.001	0.0020	<0.001
**log(Age + 1) · R (β_3_)**							
Estimate	0.0552	-	-	-	-	-	-
S.E.	0.0182	-	-	-	-	-	-
*p*-value	0.0029	--	-	-	-	-	-
**Age^2^ (β_4_)**							
Estimate	-	-	-	-	0.0001	-	-
S.E.	-	-	-	-	0.00002	-	-
*p*-value	-	-	-	-	<0.001	-	-
**THI (β_5_)**							
Estimate	-	−0.0023	−0.0031	−0.0031	0.0065	0.0110	-
S.E.	-	0.0009	0.0013	0.0009	0.0018	0.0095	-
*p*-value	-	0.015	0.018	0.002	<0.001	0.2470	-
**Random effects**							
**Lamb ID**							
Variance	0.0032	0.0012	0.0008	0.0008	0.0047	0.0696	0.0659
SD	0.0563	0.0343	0.0281	0.0280	0.0686	0.2638	0.2567
**Residual**							
Variance	0.0074	0.0026	0.0032	0.0036	0.0050	-	-
SD	0.0859	0.0506	0.0567	0.0596	0.0710	-	-

S FEED: solid feeding; RUM: rumination; MOV: moving; STA: standing; LYI: lying; GR O: grooming others; GR M: grooming mother.

## Data Availability

The data presented in this study are available on request from the corresponding author.

## Supplementary Materials

The following supporting information can be downloaded at: https://www.mdpi.com/article/10.3390/ani16050816/s1, Table S1. Chemical composition of the feeds (%DM) expressed as mean ± standard deviation. Table S2. Parameter estimates of the LMM fitted to lamb weight. Figure S1. Growth model for lambs reared Indoor and on pasture, from birth to slaughter. Gray dots: observed weights. Blue line: linear model. Table S3. Age and weight (mean ± SD) of the lambs (Indoor group, Pasture group) during the study period.
