# Supplementary material for: Early-Life Behavioral Time Budgets of a Local Dairy Sheep Breed in Indoor and Pasture Systems
Source: Animals (Basel). 2026 Mar 5;16(5):816. doi: 10.3390/ani16050816 (PMC12985204; doi:10.3390/ani16050816)
Supplement: Supplementary file 1 [file animals-16-00816-s001.zip › Supplementary_file_2.pdf]

Supplementary Material 2.

Table S2. Parameter estimates of the LMM fitted to lamb weight.

	Coefficient estimate	Standard error	t value	P value
**Intercept**	6.453	1.162	5.552	<0.001
**Age**	0.182	0.010	17.541	<0.001
**Rearing system (Pasture)**	-0.022	1.452	-0.015	0.988

Figure S1. Growth model for lambs reared indoor and on pasture, from birth to slaughter. Grey dots: observed weights. Blue line: linear model.

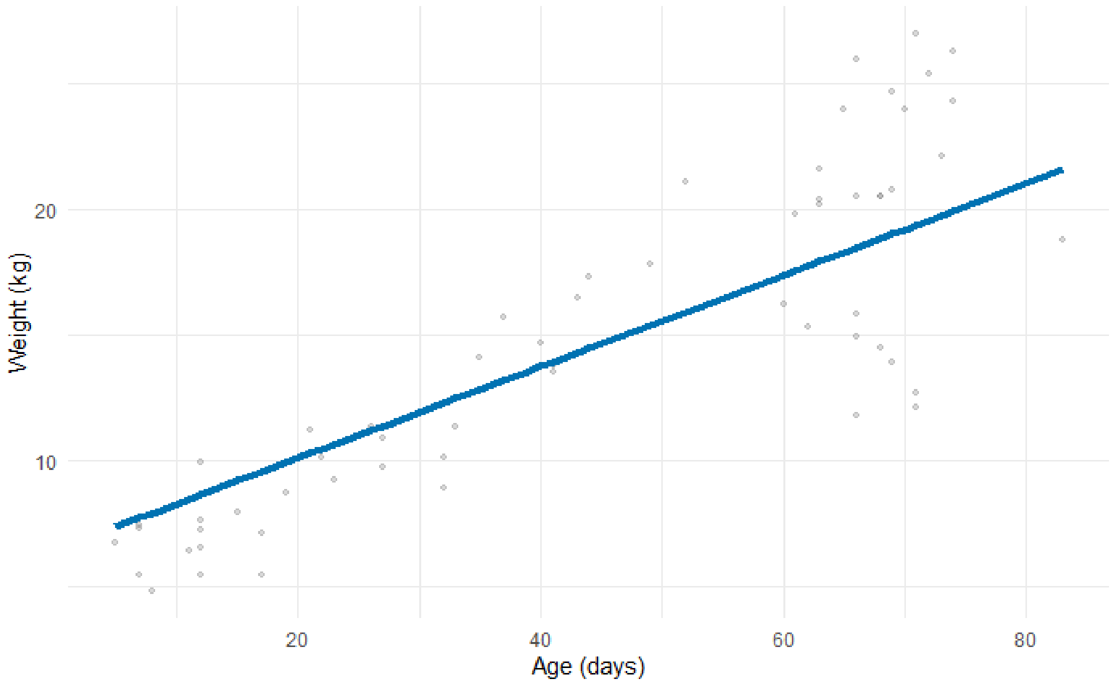

Table S3. Age and weight (mean ± SD) of the lambs (Indoor group, Pasture group) during the study period.

	**Indoor**		**Pasture**	
**Weighing**	**Age**	**Weight**	**Age**	**Weight**
**1**	13.1 ± 9.5	8.5 ± 2.9	27.8 ± 16.8	10.9 ± 5.7
**2**	47.2 ± 28.6	15.1 ± 5.8	45.8 ± 19.8	16.2 ± 6.9
**3**	36.0 ± 3.6	13.4 ± 1.8	68.8 ± 2.2	18.2 ± 5.5
**4**	67.3 ± 5.1	19.8 ± 3.5		
