# Supplementary material for: Early-Life Behavioral Time Budgets of a Local Dairy Sheep Breed in Indoor and Pasture Systems
Source: Animals (Basel). 2026 Mar 5;16(5):816. doi: 10.3390/ani16050816 (PMC12985204; doi:10.3390/ani16050816)
Supplement: Supplementary file 1 [file animals-16-00816-s001.zip › Supplementary_file_1.pdf]

Supplementary Material 1.

Table S1. Chemical composition of the feeds (%DM) expressed as mean ± standard deviation.

**Parameter**	**Hay**	**Concentrate**	**Pasture**
Dry matter	92.2 ± 1.13	93.2 ± 0.17	30.54 ± 3.67
Crude protein	14.32± 2.01	17.18 ± 0.88	15.48 ± 1.43
Ash	8.36 ± 4.00	6.07 ± 0.70	6.19 ± 2.90
Ether extract	1.61 ± 0.61	4.51 ± 0.96	2.20 ± 1.29
Neutral detergent fibre (NDF)	57.36 ± 3.76	32.93 ± 3.01	55.06 ± 5.24
Acid detergent fibre (ADF)	39.21 ± 1.88	23.11 ± 1.13	35.18 ± 3.83
Acid detergent lignin (ADL)	4.41 ± 3.72	3.82 ± 0.71	3.04 ± 1.76
